# A Study of Student-Teachers' Emotional Experiences and Their Development of Professional Identities

**DOI:** 10.3389/fpsyg.2021.810146

**Published:** 2022-01-25

**Authors:** Zehang Chen, Yin Sun, Zhenhui Jia

**Affiliations:** School of Foreign Languages and Literature, Beijing Normal University, Beijing, China

**Keywords:** teacher emotions, professional identity, student-teachers, teacher development courses, emerging identity

## Abstract

A reciprocal relation has been identified between teacher emotion and teacher professional identity. However, the underlying mechanism explaining this complex interaction remains underexamined. Moreover, limited attention has been paid to the emotional dimension of student-teachers' development of professional identity during university coursework. To bridge this gap, the present study explores how student-teachers' emotions reciprocally interact with their professional identities, drawing data from questionnaires, reflections, and interviews with students taking courses related to language teaching in a teacher-training university. Both quantitative and qualitative data delineated the intertwined trajectories of student-teachers' emotional experiences and the development of professional identity in the learning process of becoming teachers. Mainly triggered by course-related factors, student-teachers experienced a wide array of emotions, of which the polarity and intensity were determined and mediated by their goals and actions deriving from their professional identities. Those aroused emotions, in turn, signaled the developmental process of professional identity and promoted or hindered their emerging identities. This paper concludes with some implications for initial teacher education programs.

## Introduction

Teacher emotions, standing at the heart of teaching (Hargreaves, 1998), have significant impacts on the teaching and learning process (Chen, 2019). Though research on teacher education has, for decades, been solely focused on rationality and cognition (Day, 2014), scholars have recently argued for considering the affective domain (Hargreaves, 1998; Zembylas, 2004; Chen, 2021). In initial teacher education (ITE) programs, as student-teachers learn to teach, they will be experiencing a variety of emotions, which can influence their learning of professional knowledge and pedagogical skills, their understanding and commitment of the teaching profession, and their wellbeing at the university (Yuan and Lee, 2015; Birchinall et al., 2019). Scrutiny of student-teachers' emotional experiences can contribute to an understanding of how they learn to teach and become teachers, eventually informing the practices of teacher education (Sutton and Wheatley, 2003; Chen, 2021).

Teachers' professional identities, serving as a lens to explore the complexities of teaching (Beauchamp and Thomas, 2009) and teacher preparation (Luehmann, 2007), are reciprocally interrelated with teacher emotions (Rodrigues and Mogarro, 2019). While the interaction between in-service teachers' emotions and professional identities is well-documented from several theoretical perspectives (e.g., Wolff and De Costa, 2017; Yang et al., 2021), especially in the context of educational reform (e.g., Lee and Yin, 2011; Jiang and Zhang, 2021), very few studies have examined how student-teachers' emotional experiences shape their professional identities and how professional identities, in turn, influence their emotional reactions. Results from existing studies have indicated that student-teachers' emotions can largely influence their learning (Anttila et al., 2017) and their professional identities will be brought into their future teaching (Abednia, 2012). Although some studies have tackled this in the context of teaching practicums (e.g., Timoštšuk and Ugaste, 2012; Hayik and Weiner-Levy, 2019), the other half of ITE, course-taking at university, remains under-investigated. It is in teacher development courses that student-teachers, for the first time, learn formally and systematically about theoretical knowledge for teaching, which serves as critical resources for their construction of professional identity (Yuan and Lee, 2015). Identity clashes seem inevitable since student-teachers enter programs with prior experiences and differing personal values (Abednia, 2012). Additionally, they may encounter a variety of challenges in the learning and application of theory, lesson design, and micro-teaching. These are all likely to lead to the turbulence of student-teachers' emotions and the formation of professional identities.

Existing studies generally used semi-structured interviews (Chen, 2016; Hanna et al., 2019) to offer a deep understanding of individuals' emotional experiences and identity construction. To capture a more comprehensive picture, the present study adopts multiple data collection methods including focused group interviews, reflections, and questionnaires. The integration of quantitative and qualitative data helps to draw a more inclusive understanding of student-teachers' emotional trajectories and development of professional identities during their coursework at university, enriching our knowledge about the factors that may influence these dynamic processes, and eventually shed light on their complicated and reciprocal interaction.

## Literature Review

### Teacher Emotions

Given the complex nature of emotions, a consensual definition of teacher emotions has not yet been reached (Chen, 2021). In educational emotions literature, various definitions were proposed as researchers adopted different theoretical standpoints, such as from psychodynamic, sociocultural, or interactionist perspectives (Zembylas, 2007). This study, taking an integrative perspective, regards teacher emotions as “socially constructed, personally enacted ways of being that emerge from conscious and/or unconscious judgments regarding perceived successes at attaining goals or maintaining standards or beliefs during transactions as part of social-historical contexts” (Schutz et al., 2006, p. 344). This emphasizes two characteristics of teacher emotions.

The first is that emotions are evaluative and relational in nature (Zembylas, 2004). Scholars generally conceptualize teacher emotions as a multi-componential process where one critical sub-process is appraisal, a process involving the interpretation and judgment of events and situations (Sutton and Wheatley, 2003). The appraisal process starts with a phase of emotional response, where teachers evaluate their experiences in terms of goal congruence and goal relevance (Lazarus, 1991). The polarity of emotions is decided by goal congruence. Positive emotions arise when the experience is in congruence with the teacher's goals. When it is not, negative emotions arise. The intensity of these emotions is largely determined by the extent to which an encounter is relevant to the teacher's goals: high-intensity emotions will be triggered if the events matter to the teacher. The intensity of emotions can also be influenced by the appraisal process in the coping phase. Teachers' judgment of their control over events, their potential to handle events, and their expectations of events can mitigate the intensity of their emotions (Ben-Ze'ev, 2000).

The second characteristic of teacher emotions is that emotions are situated in specific contexts and evolve over time (Zembylas, 2003a; Meyer and Turner, 2006). The emotional process operates on both intrapersonal and interpersonal levels (Fried et al., 2015). On the one hand, emotions are internal feelings of individuals in relation to certain events or situations, involving biological (e.g., physiological change, emotional expression) and psychological processes (e.g., appraisal) (Sutton and Wheatley, 2003; Corcoran and Tormey, 2012). On the other hand, emotional experiences are placed within social, cultural, and political contexts, shaping and being shaped by the social relationships and values in teachers' families, schools, and cultures (Zembylas, 2003b). According to Lazarus, teacher emotions are generated neither by the teacher nor the environment but arise from the teacher-environment transactions that change over time and across different situations (1991). They are not private, universal, or stable because they are sensitive to the “lived experiences” and are “inseparable from actions and relations” (Boler, 1999, p.2). For example, in Kim and Young's study of Korean student-teachers' emotional experiences in American institutions, many negative emotions were triggered by the culturally different norms in Korean and American academic environments when the student-teachers first entered the teaching field. Afterward, those negative emotions were transformed into positive emotional rewards (e.g., pride) as some student-teachers embraced local cultural norms (2020).

The emotional needs, labor, and work required of teachers are significant and negatively affect teachers' well-being as well as the teaching workforce. The teaching profession nowadays is characterized by a high level of emotional exhaustion and burnout (Chang, 2009), highlighting an agenda to foster teachers' awareness and capacities in reflecting on and regulating their emotions. This is also an essential issue in ITE curriculums since student-teachers encounter many emotional challenges as they learn to become qualified teachers. As it is revealed in the literature, student-teachers experience many negative emotions such as anxiety, frustration, guilt, shock, and anger due to their lack of pedagogical content knowledge, unequal power relationships between student-teachers and their mentors, and hidden emotional rules embedded in field schools, local politics, and cultures, etc. (Rinchen et al., 2016; Yuan and Lee, 2016; Hayik and Weiner-Levy, 2019). Student-teachers' abilities in identifying, monitoring, and regulating their own and others' emotions are found to be important for their wellbeing, engagement in learning, academic achievement, interpersonal relationships, course satisfaction, psychosocial development, and successful classroom teaching experiences (Turner and Stough, 2020).

According to Fried et al.'s (2015) Teacher Emotion Model, teacher emotions connect the intrapersonal and interpersonal aspects through five functions: informing (providing information about oneself and the environment, thus contributing to the transformation of teacher beliefs, identities, and self-efficacy) (Dicke et al., 2015; Kalaja et al., 2016; Yuan and Lee, 2016), motivating (serve as sources of agency and influence personal motivation) (Berkovich and Eyal, 2017), giving quality to experience (subjective experiences of emotions. e.g., repeated incongruence between internal experience and external expression of emotion may lead to emotional exhaustion and teacher burnout) (Chang, 2009), influencing cognition (e.g., negative emotions may weaken working memory) (Linnenbrink and Pintrich, 2002), and regulating (e.g., regulate others' emotions) (Izard, 2010). These five functions, alone or in combination, enable teacher emotions to enhance or inhibit teaching and student learning. Furthermore, teachers' abilities to regulate their emotions are critical in upgrading the quality of instruction and easing emotional exhaustion and burnout (Lazarus, 2006; Chang, 2009). Effective coping strategies to regulate emotions are generally task-focused and emotion-focused (Lazarus, 2000). The former aims at taking actions to change the reality thereby transforming the emotions, while the latter aims to mitigate the aroused emotions through reappraising the events and situations in terms of their importance to goals and their potential to be addressed (Chang, 2009), being proactive with possible challenges and adopting a positive pattern of appraisal (Greenglass, 2002), and regulating the action tendencies of emotions (Sutton and Wheatley, 2003). These two types of coping strategies facilitate each other and work better in combination (Lazarus, 2006), though task-focused coping strategies require more motivation and agency.

### Teachers' Professional Identities

A teacher's professional identity is how a teacher defines his/her professional roles (Lasky, 2005), indicating their degree of willingness to work in the teaching profession, and reflecting their conception of “what makes a good teacher” (Qoyyimah et al., 2020, p. 7). It provides the teachers with a frame to interpret and evaluate experiences and situations, and to build ideas of “how to be, how to act, and how to understand their profession and place in society” (Sachs, 2005, p. 15). Serving as a basis for decision and meaning-making processes (Burke and Stets, 2009), teachers' professional identities are used to “explain, justify and make sense of themselves in relation to others, and to the world at large” (MacLure, 1993, p. 311). Previous studies suggest that a teacher's professional identity is dynamic and changes over time under the impacts of both individual and contextual factors (e.g., Zembylas, 2003a; Sachs, 2005). Its development is a process of integrating the teacher's existing knowledge, beliefs, and values with professional demands from ITE programs, schools, and beyond (den Brok et al., 2013). Rodrigues and Mogarro further explain that embedded in a professional community, the teacher's professional identity development involves the formation and negotiation of various sub-identities through an ongoing process of interpretation and reinterpretation of experiences within a specific culture and context (2019). The complex interactions between teachers' professional experiences and the wide range of contextual factors make teachers' professional identity intellectual and rational (Stenberg, 2010; Yuan and Lee, 2015), social and political (De Ruyter and Conroy, 2002; Hayik and Weiner-Levy, 2019), and emotional (Zembylas, 2003b; Beauchamp and Thomas, 2009) in nature.

Burke and Stets' Cybernetic Identity Model (2009) explains the developmental mechanism of teachers' professional identity. They believe identity is a “set of meanings attached to the self” (p. 50), serving as a reference for actions. When teachers' perceptions of self-relevant meanings from events or situations fail to match with their self-meanings of identity, they may take actions to change the reality for the realization of identity-verification, and the possibility of teachers taking actions is moderated by their commitment to the identity. More efforts are made when teachers are more committed to their identities (Burke and Stets, 2009). In other words, the teacher's identity system has an intrinsic need to align externally perceived self-meanings with internal identity-meanings, thus serving as a source of agency that enacts actions to make changes. During the process of identity negotiation and reconstruction, teacher agency as a driving force of action emerges salient, since external factors (e.g., educational reform, demands from teacher education institutions) alone are not strong enough to change teachers' identities (Sexton, 2008). By exercising their professional agency, teachers can actively incorporate different experiences and resources to change situations and bring external self-meanings in line with their internal identity meanings (Sexton, 2008).

Research on the shift in professional identity of new teachers highlights the necessity to raise student-teachers' awareness of identity development and prepare them for future changes in their professional identity (Freese, 2006; Thomas and Beauchamp, 2007), which naturally leads to the research aim of examining student-teacher's professional identity development in ITE programs. As they take teacher preparation courses, participate in teaching practicums in field schools, and imagine themselves as future teachers, student-teachers inevitably experience a variety of shifts, and even clashes, in their professional identity development (Beauchamp and Thomas, 2009; Deng et al., 2018; Kim and Young, 2020). Student-teachers' professional identity is found to be positively correlated with their academic performance. Those with strong professional identities react to profession-related events more positively and are more proactive in surmounting learning difficulties and overcoming poor learning conditions, compared to those with weak professional identity (Abednia, 2012; Wang et al., 2018). Therefore, it is of significance to study the developmental process of student-teachers' professional identity from a variety of diverse perspectives, including that of emotions.

### Teachers' Emotions and Professional Identity Development

Teachers' emotional experiences and professional identity development are “inextricably linked, informing each other, and redefining interpretations of each other” (Zembylas, 2003b, p. 223). Previous studies conclude that teacher emotions can shape their professional identity and mirror the developmental process (Hayik and Weiner-Levy, 2019; Rodrigues and Mogarro, 2019). For example, in Meyer's (2009) study of the interaction between student-teachers' emotional practices and professional identity, the participants built a strong emotional attachment with students as they constantly interacted with them, providing scaffolding and care. These positive emotions facilitated their emerging identity as a teacher, which was then impeded by their negative emotions derived from the lack of trust and support from their school mentors. Teachers' professional identity, in turn, can influence their emotional experiences as well. Deng et al. (2018) examined the emotional trajectory of student-teachers' professional identity formation during the practicum. A range of emotions was triggered by the dilemmas they encountered in field schools, which resulted from the conflicts between their professional identity and their actual experiences on site. A similar phenomenon was reported in Yuan and Lee's (2016) narrative inquiry of one student-teacher's emotional experiences in his practicum. The participant reported strong feelings of guilt when he followed his school mentor's instructions and requirements, which contradicted his professional identity as a student-centered teacher. Despite this, the participant experienced feelings of joy and pride when he exercised his agency within the scope allowed by the school rules to practice student-centered teaching. Previous research has fully described how teachers' emotional experiences and professional identity are intertwined and how internal and external factors may contribute to this complex interaction, but the underlying mechanism that can explain why these two constructs influence each other still requires further exploration.

Teachers' emotions and professional identity are context-sensitive. In ITE, relevant research is primarily conducted in the context of a practicum, during which a wide range of external factors can affect the interplay of emotion and identity. For instance, at the classroom level, some issues related to students and teaching activities are described as sources of joy and contentment and stimulate positive attitudes toward the teaching profession and bolster their professional identity development (e.g., Meyer, 2009; Timoštšuk and Ugaste, 2012). At the school level, hierarchical power relationships between student-teachers and their school mentors, and constant emotional labor resulting from school policies and its hidden emotional rules, lead to escalated negative emotions and diminish the development of professional identity (e.g., Yuan and Lee, 2016; Teng, 2017). At the society level, culturally specific elements in relation to the teaching profession can also shape student-teachers' emotional experiences and the development of professional identity. For example, social problems activated strong negative emotions of Arab student-teachers and restricted their professional identity development (Hayik and Weiner-Levy, 2019). In Kim and Young (2020) study, prospective foreign language teachers' native proficiency of the target language rendered them a confidence and sense of authority, while the culturally different norms in the academic environment caused anger and irritation, and inhibited identity development. The intertwined relations of student-teachers' emotions and professional identity in practicum are well-narrated from diverse perspectives, but the equally important process of initial teacher education is still under-explored.

Existing studies of teachers' emotions and professional identity mainly adopt the qualitative paradigm, using instruments like semi-structured interviews, reflections, and observations, etc. (Chen, 2016; Hanna et al., 2019). Though data collected with these measurements can provide a deep, thorough, and vivid understanding of the topic, the findings are hard to generalize beyond the involved participants, especially when the two constructs, emotion, and identity, are sensitive to context. Therefore, researchers have developed a variety of quantitative instruments to bridge the gap. To assess teacher emotions, three self-reporting instruments have recently been developed and validated. Frenzel et al.'s (2016) Teacher Emotions Scales, measuring only three discrete emotions, seem inadequate to capture teachers' diverse emotional experiences. Though Chen's (2016) Teacher Emotion Inventory includes more emotions, it takes institutional, social, cultural, and political factors into consideration, which are not directly related to student-teachers' emotional experiences in university coursework. Burić et al.'s (2018) Teacher Emotion Questionnaire, with a focus on teachers' emotions in relation to classroom teaching, is more suitable for student-teachers. Moreover, consisting of six emotions, namely, joy, pride, love, anger, hopelessness, and exhaustion, it can offer a more comprehensive understanding of student-teachers' emotional experiences. According to Hanna et al. (2019), various instruments have been developed to measure teachers' professional identity. The majority consists of multiple components, such as self-image, motivation, commitment, self-efficacy, etc. (e.g., Hong, 2010). Only a few are unidimensional. Hasegawa and Kudomi's (2006) questionnaire, focusing on teachers' understanding of teaching and the teaching profession, is compatible with our definition of teachers' professional identity.

In short, the reciprocal interactions between teacher emotions and professional identity are increasingly recognized and described in a qualitative approach, but the underlying mechanism of how teacher emotions facilitate or inhibit the development of their professional identities is left unexplained, and vice-versa. Moreover, relevant research on student-teachers mainly focuses on the teaching practicum, and their learning experiences at university seem to be neglected. Therefore, the present study intends to take multiple data collection methods and focus on student-teachers' emotional trajectory and professional identity development in the process of learning to become qualified teachers. The research questions are as follows:

(1) What are the trajectories of student-teachers' emotional experiences and development of professional identity while taking teacher development courses?
(2) What are the interactional mechanisms between student-teachers' emotions and professional identity?

## Methods

### Participants

The present study was conducted at a top-tier teacher-training university in China. The participants were recruited from a four-year pre-service English teacher education program, where a range of teacher development courses, such as Language Learning Theories, Curriculum and Teaching Materials, English Language Teaching (ELT) Methodology, Action Research for Teachers, and a teaching practicum, is offered aiming at cultivating qualified English teachers for secondary schools throughout China. In the first 3 years, student-teachers are offered theories related to ELT and engaged in practical activities like lesson design and micro-teaching. In the final year, student-teachers are required to complete their teaching practicum.

An invitation to participate in the study was sent to all second and third-year student-teachers in the program. By the end of the courses, 50 copies of questionnaires and 20 reflections were collected, among which six student-teachers were invited to participate in focused group interviews with their consent forms obtained. The six participants, all Chinese females, had taken at least two teacher development courses and experienced lesson designing and micro-teaching. They were available for participation and voluntary. Moreover, their reflections showed differing attitudes toward being an English teacher in the future. The data collected from the questionnaires and reflections provided the ability to capture the overall trajectories of student-teachers' emotional experiences and the development of professional identity in the process of learning to become teachers. The focus group interviews allowed us to integrate an in-depth understanding of their emotions and teacher identity transformation.

### Instruments

#### Questionnaires

Two questionnaires were used in this study. To illustrate an overall picture of student-teachers' emotional experiences during their university coursework, the Questionnaire of Student-teachers' Emotions (QSE) (see Appendix I) was developed based on Burić et al.'s (2018) Teacher Emotion Questionnaire. The first section of QSE collected participants' background information including gender, grade, and courses taken. The second section, consisting of 19 five-point Likert items ranging from one (disagree) to five (agree) and two multiple-choice items, aimed to capture participants' emotional experiences and possible influencing factors. We reworded the original items based on student-teachers' experiences pertinent to teaching during their coursework. For example, “My students evoke feelings of love inside me.” was adapted into “Thinking of my future students evokes feelings of love inside me.” The Cronbach's alpha coefficient of positive emotions and negative emotions was α = 0.873 and 0.778 respectively. In addition, Hasegawa and Kudomi's (2006) Professional Identity Questionnaire, consisting of 17 five-point Likert items ranging from disagreeing to agreeing, was adopted to measure student-teachers' perceptions of the teaching profession. In our study, the internal consistency coefficient was α = 0.831. These two questionnaires were administered online and altogether 50 valid questionnaires were collected by the end of the semester.

#### Reflections

Learning reflections served as a critical source of data for the present study. Student-teachers were suggested to reflect on their emotional experiences during their coursework with a structured reflection sheet (see Appendix II), which aimed to elicit the strong emotions they experienced during the beginning, middle, and end of the courses. Information about factors leading to the emotions and actions taken to regulate these emotions was collected as well. All reflections were written in Chinese as the participants felt it more convenient and were more comfortable using their first language. Twenty student-teachers voluntarily submitted their reflections.

#### Focus Group Interviews

To explore the interactional mechanisms between student-teachers' emotions and professional identity, six participants were invited to participate in the focus group interviews (for interview questions, see Appendix III) after they submitted their reflections. Due to the different availabilities of participants, the interviews were scheduled across 2 days. Fang and Tan (pseudonyms) took their interview together, and Yuan, Han, Jia, and Ling (pseudonyms) were interviewed together the next day. The participants were asked to report their emotional experiences and understanding of teaching during their coursework. This was aimed at elucidating student-teachers' changes in emotions and professional identity, as well as possible factors contributing to these changes. The interviews were conducted in Chinese and audio-recorded with participants' permission. The recordings were transcribed verbatim and checked by the authors, which resulted in a text of 18,425 words.

### Data Analysis

Quantitative data drawn from the questionnaires were exported to SPSS 26 for descriptive analysis. Statistics including mean, median, range, standard deviation were calculated to explore the patterns of student-teachers' emotions and professional identity. Qualitative data collected from reflections and interviews were prepared and submitted to NVivo 12 for inductive coding. The second and third researchers first independently open coded the data and identified a range of themes and categories. They then conferenced with the first researcher to determine the final coding and eventually reached four major themes: teacher emotion (e.g., “felt confused”), professional identity (e.g., “new understanding of teacher's responsibility”), emotional regulation (e.g., “sought help from the teacher”), and learning goals (e.g., “to get prepared for future research-fund application”). Based on the data regarding emotions, emotion regulation, and changes in professional identity from reflections and interviews, the developmental trajectories of student-teachers' emotions and professional identities were delineated by critical events and supported with evidence from questionnaires, as shown in Table 1. With further analysis of learning goals and emotional regulation, the interactive mechanisms underlying them were revealed.

**Table 1:** The integration of data sets.

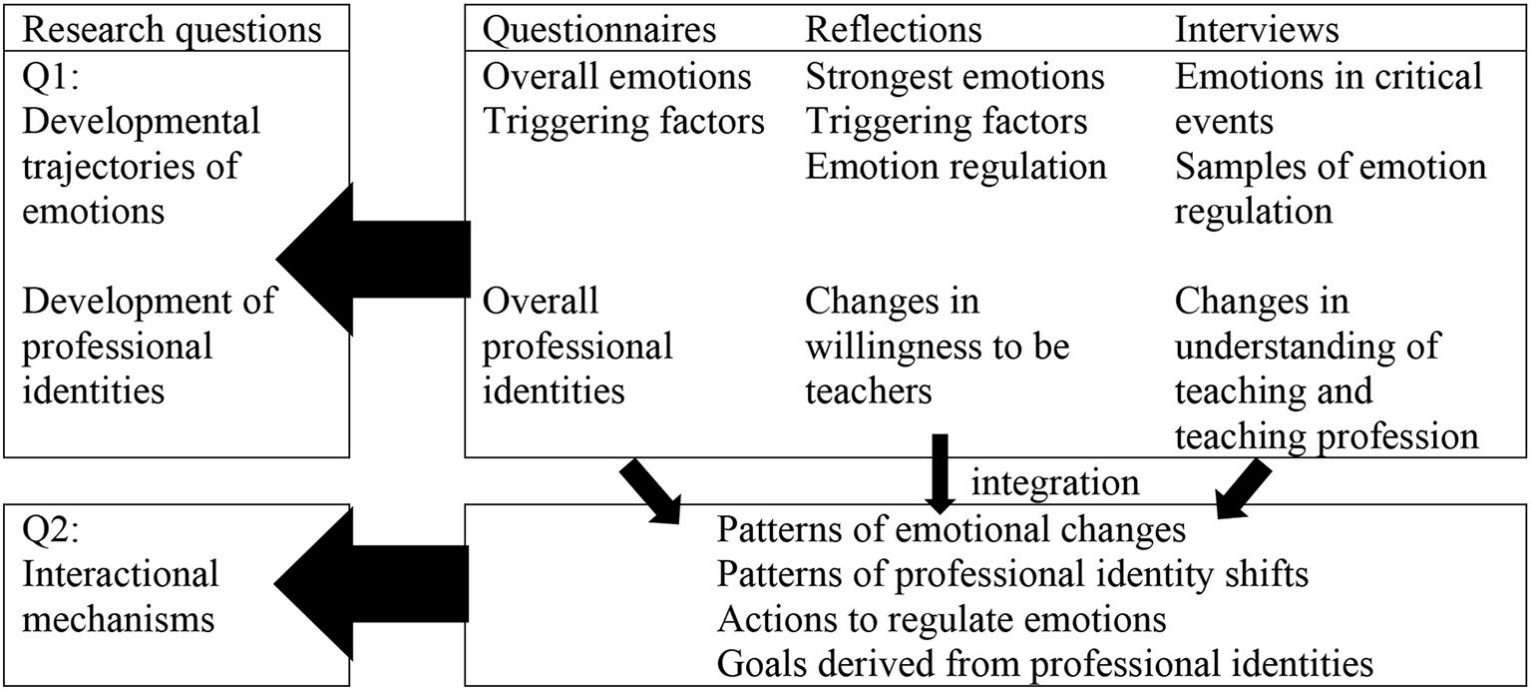

## Findings

The analysis revealed the intertwined trajectories of student-teachers' emotional experiences and professional identity development. This section presents the emotions and professional identity transformation the participants experienced during their coursework.

### Enlightenment and Confusion: Clashes Between Original Identities and Designated Identities

The prior learning experiences and perceptions of English teaching that student-teachers possessed shaped their original teacher identities. In the process of learning to be teachers, ongoing negotiation occurred between student-teachers' original identities and learned pedagogical knowledge and skills, of which some were accepted, leading to intense feelings of enlightenment and construction of designated identities prescribed by experts. In general, the participants tended to feel excited when they were learning (M = 3.64) and applying (M = 3.70) language learning and teaching theories because the professional knowledge had changed their views about English teaching and helped them better understand the teaching profession:

I find this lesson (Language Learning Theories) very useful. My current teaching is based on how I was taught in high school, and I will simply replicate the methods my teachers used without considering if they are scientific or effective. However, language learning theories tell us whether the method is theoretically effective or not… After learning those theories, I believe there are many possibilities in teaching apart from the methods my teachers used (Yuan, Int02-A07).

However, apart from feelings of enlightenment, emotions like confusion and distress were also reported. Although student-teachers were suggested to apply the newly learned ELT methods, they were not completely convinced that these methods would work and they needed more time to digest and internalize them. Their sense of insecurity was aroused due to the inconclusive nature of theories in language learning and teaching. There are multiple approaches to English teaching with theoretical underpinnings in literature, yet scholars in the academic community have not reached a consensus regarding effective English teaching. Additionally, student-teachers felt overwhelmed when asked to learn a mass of knowledge within a limited time.

There are some inconclusive theories in the course (Language Learning Theories). When someone proposes a theory, others will critique its disadvantages. That's to say, you need to make your own decisions while teaching (Ling, Int02-A154).

I think this course was a little messy at the end. First, the content in every lesson was too much for us to digest. Second, the teacher often asked questions about the theories we learned before, but few students can answer (Ling, Int02-A157).

The participants tried to link their learned knowledge to their prior English learning experiences, but the instructional methods introduced by the teacher educator were found to be markedly different from the way they learned English. Moreover, according to their past learning experiences, to succeed in the College Entrance Examination (CEE), practicing language knowledge and skills such as grammar and reading comprehension was considered more important than fostering students' overall competencies. The student-teachers felt confused facing the dilemma of following the guidance of the school curriculum or following the “practical needs” resulting from preparation for the CEE. Even if they would like to adopt new approaches, they were worried about the attitudes of various stakeholders.

I felt confused. The new curriculum standards and professors all ask teachers to foster students' core competencies, but in the context of exam-oriented education, school leaders, parents, and students will not cooperate. Teachers cannot implement that type of instruction despite their willingness to do so. They are strong in will but weak in power (Yuan, Int02-A190).

Many participants questioned the practicality of applying the learned knowledge in their future teaching based on their prior learning experiences. Thirty-six participants out of 50 (N = 36/50) reported feeling hopeless when they couldn't apply learned theories. Additionally, they found learned methods were often too complicated and exhaustive to be applied in daily teaching, which led them to question the necessity of learning such teaching methods.

If teachers plan every lesson this way, the workload is heavy. It is incongruent with my experiences. Many teachers don't even write lesson plans in advance (Wang, Ref-D16).

We discussed this in private. We thought this method was good, but our daily lessons would not be like this if we were high school teachers. Generally speaking, it appears only in demonstration lessons. It seems not that useful even if we are high school teachers in the future, given the context of exam-oriented education (Fang, Int01-B35).

### From Anxiety to Contentment: Constructing the “Virtue-Fostering Educator” Identity

Different identities resulted in different attitudes of student-teachers toward courses. The majority held positive attitudes because they were “Government-funded Normal Students^1^” which meant that they were likely to be teachers in the future due to the requirements of the financial support. They shared feelings of expectation and excitement due to their beliefs that teacher development courses are helpful for their future careers. However, some participants do not wish to become teachers and a few felt they were being forced to take these courses:

My strongest emotion during the coursework was expectancy because to be a high school English teacher is my dream. Therefore, I expected these elective courses related to English teaching could teach me something useful (Deng, Ref-D46).

At the beginning of this course (English Teaching design), I felt bored and resisted it from the bottom of my heart, because I am not a “Normal Student” and I didn't want to take this course. However, I had to take it because of the credit (Fang, Int-B03).

Guided by the latest version of *English Curriculum Standards for Senior Middle Schools* (Ministry of Education., 2018), this program aims to prepare secondary school English teachers to be capable of fostering students' virtues through English education. It is characterized by the integration of instrumentality and humanity of English and emphasizes the comprehensive cultivation of students' core competencies. As student-teachers learned to design lessons that cultivate core competencies and foster virtues, they gradually recognized and acknowledged the multiple goals of English education in China, and transformed this understanding into their lesson-design practices.

After learning the course, I have developed a new understanding of lesson design and teachers' responsibilities… The teachers are responsible to foster virtue through education, which is quite challenging because knowledge can be taught, but virtue requires nurturing with love and care… These all made me realize the huge responsibilities and requirements of this career (Lee, ref-D59).

In this process, student-teachers were slowly constructing a virtue-fostering educator identity and at the same time experienced plenty of negative emotions which were detrimental to their professional identities. This was mainly because of the difficult course content, heavy-loaded course assignments, and course assessment policy. Among all of their workload tasks, lesson design was one of the most challenging tasks for student-teachers, and the repeated processes to improve their designs were even harder and more exhaustive. Furthermore, lesson design was a critical part of course assessment, but participants' self-reported preparedness for lesson design was clustered around 3 (M = 2.96, SD = 0.82), indicating their low confidence in designing lessons. These all contributed to their feelings of anxiety and lower willingness to be teachers:

Lesson design is very complicated…I devoted a lot of time and energy into the design for the fear of a bad outcome. This made me exhausted…Seeing other students perform well in the design made me more anxious… With those emotions, my willingness to be an English teacher at primary or secondary schools was lower (Lee, Ref-E59).

I felt anxious because assignments and lesson design were a critical part of course assessment. I spent a long time on lesson design… (Guo, Ref-D22).

In bearing these negative emotions, some participants took actions to regulate them and ease the negative influences on their lives and studies. The majority chose to act and solve the problems that had caused the anxiety. For instance, Hang felt extremely anxious and confused because he found it hard to design lessons that could foster virtues and core competencies. Facing the task of designing a lesson, his mind “went blank” and he was too anxious to get started. To regulate those emotions, Hang analyzed the lesson designs shared by the teacher educator and classmates and documented the “surprising, creative, interesting” ideas for future reference. In this way, he tried to improve his ability in lesson designing and move closer toward his virtue-fostering goals. His growth thus eliminated the negative emotions and activated positive ones. Instead of taking actions to change a dissatisfaction with reality, some participants chose to take a positive attitude, comforting and encouraging themselves. A few, on the contrary, suppressed their negative emotions.

It is noteworthy that not all participants reported a reduction in teacher identity after the challenging tasks of lesson design. Some student-teachers believed it was good to expose problems early because this would benefit their growth as preservice teachers. A few revealed that the challenging nature of being a teacher changed their minds and started the construction of teacher identity:

My performance was not that satisfying, though I am still filled with hope instead of anxiety. I think it's good to expose problems early and I won't deny myself because of it. I can face my shortcomings more calmly with feelings of hope and confidence (Ran, Ref-D67).

Before taking this course (English Teaching Design), I didn't want to be a teacher because I thought it was not challenging. It is believed to be a good job for girls for its long vacations and unchallenging nature. However, after this course, I found it requires lots of effort to teach well… I now want to be a teacher because it's not easy. It's challenging (Ling, Int02-H173).

At the end of the semester, the participants generally experienced a sense of achievement. The fact that student-teachers felt content seeing their growth in designing virtue-fostering lessons indicates their acknowledgment of the effectiveness of this approach to some degree. Therefore, the sense of achievement signaled the initial internalization of their virtue-fostering educator identity:

I can see my progress every time I complete a task. I started from a novice not being able to design any activity and turned into someone who can design a complete lesson…I felt my gains and achievement in this course (Dong, Ref-D04).

The feelings of contentment also increased student-teachers confidence in teaching and strengthened their professional identities:

I can feel myself indeed making progress and knowing more about my future career. This helped me keep confident and interested in teaching (Hang, Ref-D10).

The lesson plan I designed gave me a great sense of achievement. It helped me discover this novel, unique sense of being a teacher (Han, Ref-D14).

### Frustration or Pride: Exercising the “Reflective Practitioner” Identity

After practicing lesson designing, student-teachers were assigned into groups of five or six and conducted micro-teaching based on their lesson plans. Experienced high school teachers were invited to join the groups and comment on the student-teachers' performances. The whole process was video-recorded for later reflection. With feedback from both the teacher and the peers in the group, student-teachers then reflected on their experience and tried to improve their lesson designs. While exercising the reflective practitioner identity, the participants experienced diverse emotions resulting mainly from their perceptions of teaching effectiveness and feedback from the teacher and peers.

Overall, the participants felt excited when the micro-teaching went well, such as building a positive classroom atmosphere (M = 3.36) and achieving teaching goals (M = 3.12). They were proud when their activities worked effectively (M = 4.16; N = 35/50), when they received positive feedback from the teacher (M = 4.00; N = 29/50) and peers (M = 3.92; N = 18/50), and figured out how to improve their lesson designs based on feedback and reflection (N = 26/50). These positive emotions enhanced student-teachers' interest and confidence in teaching and strengthened their professional identities:

I was not anxious anymore after completing the task. I got some positive feedback and learned a lot about English teaching. I've already started to review my micro-teaching and am trying to improve it (Kai, Ref-D51).

The teacher in our group was nice. She pointed out the shiny spots in our micro-teaching and enhanced our confidence (Ling, Int02-A177).

Micro-teaching this semester let me experience the joy of being a teacher. I planned to find an internship in educational institutions this summer vocation (Deng, Ref-D48).

Conversely, some participants encountered difficulties in practicing micro-teaching and improving their lesson designs, which led to unsatisfying outcomes and activated feelings of frustration since they had invested plenty of effort in preparation and expected a good outcome. Many participants felt hopeless when they found their activities did not work in micro-teaching (N = 31/50) or did not know how to improve their lesson designs (N = 39/50). These frustrating outcomes made them question their abilities in teaching and impaired their professional identities.

I had been working hard to prepare for micro-teaching and expected comments like “wow, wonderful” from others. However… the outcome was not what I wanted, making me frustrated and completely discouraged… Now I don't want to be a teacher in secondary schools anymore (Yuan, Int02-A51).

But interestingly, only a few participants reported frustration when they received negative feedback from teachers (N = 11/50) and peers (N = 8/50). They were more likely to feel frustrated when they were in trouble with theory application (N = 33/50), lesson design (N = 31/50), and improvement (N = 39/50). In fact, teachers and peers often provided suggestions for lesson design improvement when they pointed out the shortcomings. These helpful suggestions may decrease the intensity of frustration caused by negative feedback.

### Expectancy: The “Motivated Learner” Identity Emerges

Although the student-teachers had experienced ups and downs in their emotions and professional identities, they generally held positive attitudes toward the teaching profession and teacher preparation courses at the end of the semester. In one way, they made much progress in teaching-related competencies. In another way, they developed new understandings of the profession.

I have learned a lot of basic knowledge about teaching after taking these courses. I am full of expectations for my future career as a teacher (Ming, Ref-D54).

I believe I can improve more in the forthcoming learning. Although we have many problems, we will make ongoing progress! (Ran, Ref-D67).

The participants generally highly acknowledged the importance of ethical sense (M = 4.38), specialized knowledge and skills (M = 4.24), good interpersonal relations (M = 4.24), the spirit of self-sacrifice (M = 4.18), commitment (M = 4.12), and accountability for practical success (M = 4.06) in being an English teacher. They believed teachers can express their personality during work (M = 4.16) and connect emotionally with students (M = 4.24) and are less likely to get the job done according to their own principles (M = 3.74), but rather need to follow the rules and procedures (M = 4.04) of schools. These attitudes are all preparing them for their future teaching careers.

The participants in the questionnaire reported affection toward teaching (Mean = 3.58) and being an English teacher in the future (M = 3.48). The idea of working with students and making positive influences on them evoked love (M = 3.82) and joy (M = 4.26). However, the participants also reported feelings of anxiety due to low confidence in teaching (M = 3.52) after their coursework. They felt they have “just started” because there were still plenty of questions needing further exploration:

I think my ability in lesson design is still inadequate. Many theories learned in this course (English Teaching Design) have not been mastered and applied well. My performance in micro-teaching and the final exam did not meet my expectations. I have made some progress this semester, but these growths are still not enough (Dong, Ref-D05).

I have a sense of eagerness to learn more. The one-semester course is too short and we have only learned how to teach reading… there are still many problems unsolved, such as how to arrange the timing of activities, or how to interact with students, etc. (Lee, Ref-D61).

With the experiences of learning and practicing teaching, student-teachers, to some extent, internalized the teacher professional identity, which gave them the confidence and desire to become qualified teachers in the future. Therefore, their self-perception of inadequacy in virtue-fostering teaching, including both their theoretical knowledge and practical knowledge, yielded the need for further professional development, serving as intrinsic motivation for ongoing learning. This led to a sense of expectancy for their upcoming teacher preparation courses:

It feels like I have not learned enough. Though the (learning) process was painful, I am looking forward to the courses next semester. Hope I can learn more specific content and learn better (Jia, Int02-A121).

My preferred career is English teacher, which requires constant professional development. So, I selected the course, English Language Teaching Methodology, for next semester. Although some seniors told me this course was heavy-loaded and was the 3.0 version of English Teaching Design, I think it's necessary to take this course so that I can learn more from it (Tan, Int01-B67).

## Discussion

Using multiple methods to collect data, this study enabled us to capture a comprehensive picture of, and analyze in-depth, the interaction of student-teachers' emotional experiences and professional identity development during their coursework. Their relationship is not one-way or linear. They are essentially intertwined and reciprocally influenced by each other (Chen, 2021; Cheng, 2021). Triggered by course-related factors, student-teachers have experienced diverse emotions, of which the polarity and intensity are determined and regulated by their goals and actions deriving from existing identities (Burke and Stets, 2009). The changes in emotions signal the development of teacher identity, the positive emotions strengthen their emerging identities, while the negative emotions inhibit them (Teng, 2017).

### Emotion as an Indicator of and Catalyst for Professional Identity Development

**Figure 1:**
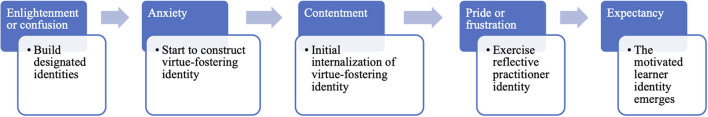
Teacher emotions as an indicator of identity development.

To begin with, student-teachers' emotions serve as an indicator, explicating the invisible process of professional identity development. Extending previous research findings that consider emotions as a mirror of or analytical lens for identity development (e.g., Reio, 2005; Hayik and Weiner-Levy, 2019), this study specifies that teacher emotions signal the critical points in the developmental process of professional identity. As shown in Figure 1, with ongoing coursework, student-teachers' emotions have tremendously changed, segmenting the developmental process into multiple sections. At the beginning of coursework, as student-teachers learn theories in relation to teaching, their ontological and epistemological beliefs about language teaching started to change, leading to the gradual construction of designated identities prescribed by teacher educators (Jiang and Zhang, 2021), which may differ from their prior learning experiences and existing ideas about English learning. Thus, a sense of enlightenment is activated when they accept the learned views and change their original understandings, indicating their original teacher identities are transformed into designated identities. Otherwise, feelings of confusion would be triggered, signaling the professional identity transformation is inhibited. Moving on to the next session, as student-teachers are learning to design virtue-fostering lessons, which is a difficult and exhaustive portion of the course as well as a part of course assessment, feelings of anxiety are provoked, signifying the starting point of the construction of virtue-fostering identity designated by the teacher educator. Facing intense negative emotions, they take actions to overcome difficulties and make progress in lesson design, during which the feelings of contentment emerge and the intensity of anxiety decreases, indicating an initial internalization of virtue-fostering identity. As student-teachers conduct micro-teaching and reflectively improve their lesson designs, the outcomes lead to feelings of either pride or frustration. This is when student-teachers exercise their reflective practitioner identities and realize the gap between them and qualified English teachers, which naturally leads to their construction of motivated learner identity. Driven by the intrinsic motivation for professional development, they are looking forward to the forthcoming courses related to teaching so that they can be equipped with profound pedagogical knowledge and professional skills for future teaching opportunities. Both student-teachers' emotions and professional identities are dynamic and evolving along with their coursework (Sachs, 2005; Deng et al., 2018), while their emotions clearly mark their changes in identities.

Serving as a catalyst, student-teachers' emotions, mediated by their self-confidence, can positively or negatively influence their professional identity development. In their coursework, diverse negative emotions triggered by external factors might undermine student-teachers' self-confidence, thus inhibit the development of their emerging identities and decrease their willingness to be teachers (Chen et al., 2020). Echoing previous research findings (Teng, 2017; Kaur, 2018), this study demonstrates the detrimental impact of negative emotions on professional identity construction. Multiple course-related factors including challenging tasks and assessment requirements leave student-teachers especially vulnerable to a wide array of negative emotions, which reduces their self-confidence, triggers self-doubt, and undermines their job motivation (Wolff and De Costa, 2017). As a result, their emerging identities are hindered and professional identities are weakened (Chen et al., 2020). For instance, bearing the idea that good lesson designs require a comprehensive understanding of curriculum, accurate interpretation of the text, and appropriate setting of teaching objectives, Lee considered the task to design virtue-fostering lessons complicated and challenging, and devoted her energy to every step of the process. However, given all the effort she expended, her performance in lesson design was still unsatisfactory. This led to her feelings of exhaustion, anxiety, and self-doubt about her ability to become a qualified teacher. Central to these feelings is her self-perception of inadequacy in accomplishing her educational goals and exercising the designated virtue-fostering educator identity. Therefore, those negative emotions Lee experienced curbed her construction of virtue-fostering educator identity and diminished her professional teacher identity.

Oppositely, the positive emotions student-teachers experience during their coursework strengthen their emerging identities and increase their desire to be teachers. This study provides empirical evidence to the view that positive emotions provoked by student-teachers' sense of achievement and progress can raise their self-confidence (Bao et al., 2021) and boost their emerging identities (Yuan and Lee, 2016; Kaur, 2018; Chen et al., 2020). Faced with the difficult and demanding assignments to design lessons and conduct micro-teaching, the student-teachers invest considerable time and effort to grow from knowing nothing to someone who can complete the tasks independently. Therefore, intense feelings of accomplishment and growth are elicited when their works are recognized or even praised by the teacher educator, experienced teachers, and peers. These feelings replace student-teachers' prior anxieties with contentment and pride, and practice the “motivate” function (Fried et al., 2015), which enhances their confidence in conducting teaching-related tasks, promotes the internalization of virtue-fostering identity and reflective practitioner identity, and bolsters their motivation to be teachers. For example, Dong was worried about her ability in designing lessons and was afraid to express ideas in group discussions or in front of the class due to low self-efficacy. She thus tried every method for improving her lesson design ability and her efforts found some success. Seeing her progress in designing virtue-fostering lessons made her “content” and “less anxious”, and increased her confidence in fostering students' virtues through English lessons, which indicated her gradual internalization of the virtue-fostering identity. These strong feelings of contentment and pride can alleviate the preceding negative emotions and compensate student-teachers' hard work by making them feel worthwhile. As Tan said in the interview, “though the learning process was painful, we can learn something useful in the end. I think this course is pretty good.”

However, negative emotions do more than simply diminish student-teachers' professional identities. As discussed in previous sections, teacher emotions signal the developmental process of professional identity. Negative emotions are generated when student-teachers encounter problems in learning how to teach, which activates the construction or reconstruction of professional identity. As exemplified in this study, feelings of confusion are triggered as student-teachers face the clashes between original identities and designated identities, dwelling on what to believe and what to do; severe anxiety is produced when they are struggling on designing and improving their lesson plans, and frustration is aroused by unsatisfied outcomes in lesson design and micro-teaching performance after a long period of preparation. As student-teachers reflectively confront the process and outcome of some emotionally engaging events, the aroused negative emotions, by exercising the “inform” function (Fried et al., 2015), diagnose and provide information about their learning and professional identity development (Winograd, 2003; Kaur, 2018). In Dong's reflection, for example, her feelings of worry informed her that problems occurred in learning and effective measures were required for further improvement. Student-teachers need to understand how their emotions affect the quality of their learning and the construction of professional identity and learn to use the emotional information to facilitate their professional development.

### Professional Identity as a Determinant of Emotional Experiences and Drive for Emotion Regulation

Situated in the context of university coursework, the external factors shaping student-teachers' emotions are different from those found in the teaching practicum. Student-teachers' relationships with their mentors and students, and the situated school system and hidden emotional rules, politics, and cultural norms are identified as critical factors in the practicum (Yuan and Lee, 2016; Hayik and Weiner-Levy, 2019; Kim and Young, 2020). During the coursework, the emotions are primarily influenced by factors related to course contents, including task difficulty, assignment load, teaching pace, and assessment. Feedback from teacher educators, experienced teachers, and peers is also an important factor. Other interpersonal factors, such as peer pressure, peer collaboration, and peer encouragement are mentioned by some participants. Furthermore, the educational context and cultural views of the teaching profession at the macro-level can impact student-teachers' emotions as well. At the university, the learning dimension emerges salient as student-teachers' are struggling with theories and lesson designs. The external factors shaping their emotions are mainly from their interaction with their courses. While in the field school, student-teachers seem to be taken as professionally qualified teachers and the influencing factors cluster around the social and cultural dimensions.

Previous research on in-service teachers has demonstrated the impacts of educational reform on teacher emotion and professional identity (Lasky, 2005; Jiang and Zhang, 2021). Student-teachers also undergo a special “reformation” while incorporating learned teaching knowledge into their existing understanding of teaching, during which clashes between their original identities and designated identities are inevitable. However, student-teachers seem to be more open to changes and inclined to change their views and feel enlightened while in-service teachers tend to react negatively or reflect self-preservation when the reform agendas are incongruent with their professional orientation (van Veen and Sleegers, 2006). This may be attributed to the complex environment in-service teachers are situated in, where exam pressure is real and all stakeholders are involved. Lee and Yin's (2011) argument for the existence of a reciprocal relationship between educational reform and teacher emotions works for student-teachers as well. Educational changes lead to emotional changes in teachers and the emotions they experience influence the ways and extent to which reforms are accepted and adopted. Student-teachers, as demonstrated in this study, experienced different emotions during the negotiation between designated identities and existing identities, and these emotions, in turn, facilitated or inhibited their emerging identities.

In terms of internal factors shaping their emotions, student-teachers' set learning goals such as being able to design and conduct virtue-fostering lessons driven by their student identities and emerging teacher identities. These goals, serving as a determinant of emotional experiences and drive for emotion regulation, explain why they experience emotions with different polarity and intensity under certain circumstances, and why they make efforts to regulate their emotions and polish their products. The polarity of emotion is determined by student-teachers' judgment of whether the situation is congruent with their goals (Sutton and Wheatley, 2003). Goal congruence leads to positive emotions and goal incongruence to negative emotions (van Veen and Lasky, 2005). In this study, negative emotions are triggered when the events or situations hinder student-teachers from improving their abilities in designing virtue-fostering lessons. For instance, feelings of confusion were aroused when the uncertainty of the most appropriate instructional method occurred while learning theoretical knowledge; anxiety was engendered when they found the lesson design was challenging and the assignments were heavy-loaded; frustration was triggered when the outcomes in micro-teaching were unsatisfying. In student-teachers' perception, these factors all impede their progress in goal realization, thus causing an array of negative emotions. Conversely, positive emotions are experienced when student-teachers learn conclusive research-based theories about teaching. They are also experienced when student-teachers make achievements and progress in designing virtue-fostering lessons and micro-teaching, which are in congruence with their goals. As Deng wrote in her reflection, “To be a high school English teacher is my dream and micro-teaching lets me experience the joy of that in advance.” Facing the demanding and challenging nature of teaching, many student-teachers experience anxiety and self-doubt, while other participants, on the contrary, start to construct their teacher identities because of their desire to work in a challenging profession. As Ling said, “I now want to be a teacher because it's not easy. It's challenging.”

In addition, the goal relevance of events is revealed to be a critical factor in determining the intensity of their emotions (Lazarus, 1991, 2000). The more relevant a student-teacher judges an event or situation, the more intense the emotional experience is (Chang, 2009). For example, the policy of including lesson design and micro-teaching as part of course assessment was reported as a major source of anxiety. Student-teachers feel anxious because the high level of difficulty in lesson design and micro-teaching prohibits them from realizing the goals of designing and conducting virtue-fostering lessons. By incorporating performance in lesson design and micro-teaching into course assessment, student-teachers' performances are closely linked to their most critical goals of achieving good scores derived from their student identities, which intensifies their feelings of anxiety. According to Guo, knowing lesson design was a part of the assessment made her anxious, leading her to spend extensive time on lesson design forgoing eating or drinking, and suffering from severe backache. Conversely, Yuan reported stress-free feelings and attributed the reason to “no assessment” since she did not register for the course. She, therefore, felt joyful rather than anxious, though the course content was overwhelming and challenging. In Yuan's appraisal, any gains from this course are bonuses, and the obstacles are not significantly detrimental for her goal realization.

While a wide array of emotions are triggered by the interaction of external and internal factors, student-teachers' actions enacted by student identities and teacher professional identities are found to be essential in shaping their emotional experiences and emerging identities. Informed by negative emotions, student-teachers notice the incongruence between reality and their goals, which provokes their agency, driven by the intrinsic need of the identity system to align reality with identity standards, in this case, goals (Burke and Stets, 2009). Therefore, the degree of agency depends on the extent to which student-teachers are committed to their goals. With a strong commitment, they are highly motivated to learn and exercise agency. They actively incorporate experiences and resources to change their reality and align it with their goals, which may mitigate and even transform their prior negative emotions into positive ones (Sexton, 2008; Jiang and Zhang, 2021). For example, Hang invested great effort to improve his ability in designing virtue-fostering lessons by analyzing the samples shared by teachers and classmates, and learning from their creative and attractive ideas. This constant effort, according to him, resulted from dissatisfaction with his first lesson design, which was “inflexible, tedious, and exam-oriented.” In Hang's appraisal, his then-current ability in lesson design was far away from his goals. Driven by the intrinsic urge to bring his ability in line with his goals, that is, being able to design virtue-fostering lessons, his agency was activated and actions were enacted to change the reality with available resources. With the growth in his ability in lesson designing, the gap between goals and reality was narrowed. Hang's emerging identity as a virtue-fostering educator is increasingly internalized and prior anxiety is gradually replaced by contentment. In other words, Hang's actions, driven by his professional identity, regulate his emotions and shape his identity development.

**Figure 2:**
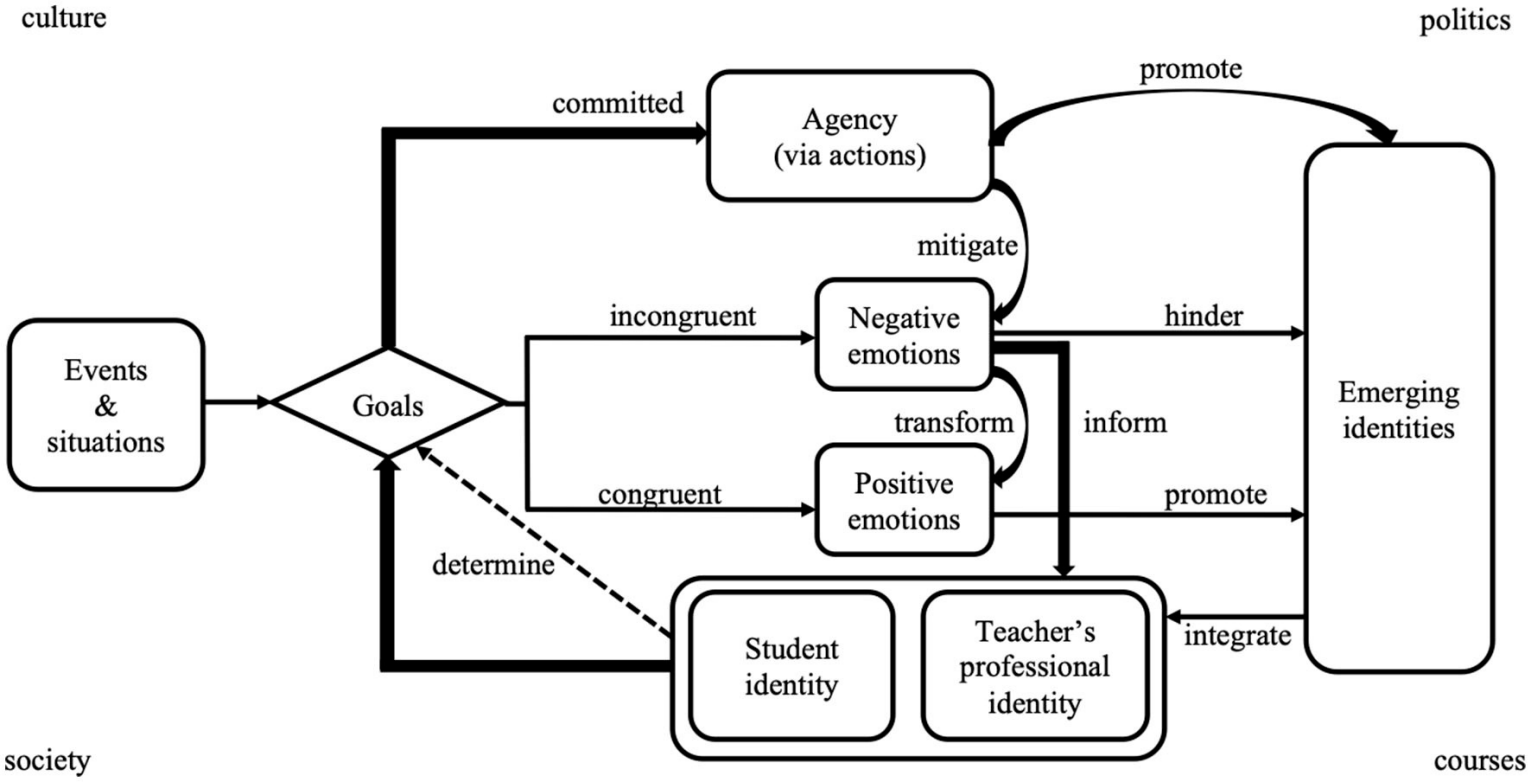
Interactional model of teacher emotion and professional identity.

In summary, student-teachers' emotions and professional identities are fundamentally intertwined and reciprocally interact with each other (Chen, 2021; Cheng, 2021) as illustrated in this study. Stimulated by external factors, including overwhelming theories, challenging tasks, heavy-loaded assignments, and assessment, student-teachers appraise the events or situations against their goals derived from their professional identities to determine the polarity and intensity of emotional reactions. If the reality is in congruence with goals, positive emotions are then triggered and facilitate the emerging identities. If the reality is not congruent with goals, negative emotions are activated and hinder the emerging identities. The provoked negative emotions inform student-teachers of the occurrence of goal incongruence and activate their professional agency if they are committed to the goals. By exercising the agency, they actively incorporate available resources to change the reality and bring it toward the goals. The more committed to the identities, the more effort student-teachers would make to align the reality with the goals (Burke and Stets, 2009). As the gap narrows, the emerging identities are gradually constructed and the prior negative emotions are regulated and transformed into positive ones. An interactional model representing this complex process is shown in Figure 2.

## Conclusions

Informed by the data collected from multiple sources, this study delineated the common developmental trajectories of student-teachers' emotional experiences and professional identities in the process of becoming a teacher. More importantly, it revealed the external factors shaping the development process and illustrated the reciprocally interactional mechanisms (Chen, 2021) between teacher emotions and professional identity in the context of university coursework. Student-teachers' professional identities determine the polarity and intensity of the emotions they experience through goals and actions, while these emotions in turn signal and shape the constructional processes of emerging identities. Based on such an understanding, two major implications are proposed for ITE programs in terms of emotion and professional identity development. First of all, as professional identity development is the process wherein student-teachers construct their own ideas and build practical knowledge (Teng, 2017), it is of importance to foreground identity negotiation in ITE programs (Kim and Young, 2020). Teacher educators need to recognize the signaling role of emotions in identity development and sensitize student-teachers to the invisible changes in identities by highlighting the role of relatively more explicit emotions. For example, student-teachers may benefit from engaging in group discussions or reflective activities when they experience feelings of confusion, triggered by the clashes between their existing identities and designated identities. With opportunities to reflect on their emotions and identities, student-teachers may be more aware of the shifts and tensions in their professional identities (Yuan and Lee, 2015). Moreover, teacher educators also need to equip student-teachers with effective strategies to grapple with the identity changes in the present and future.

Second, given the promoting function of positive emotions and inhibiting role of negative emotions on emerging identities, it is of tantamount significance for student-teachers to regulate negative emotions and transform them into positive ones. However, the strategies utilized by the participants in this study are limited to task-focused coping strategies, which are effective but time-consuming. To foster student-teachers' capacities in promptly coping with intense negative emotions, teacher educators need to help them build the awareness of regular reflection on emotional experiences (Yuan and Lee, 2016) and teach them how to use emotion-focused coping strategies to reduce the intensity of negative emotions within a short time (Lazarus, 2000). Student-teachers can regulate the intensity of emotions by reappraising the significance of the events or situations to their goals, and their potential to align the reality with the goals (Chang, 2009). Besides, at the very beginning of each course, teacher educators can inform student-teachers of the possible difficulties and challenges of the forthcoming courses. By using this proactive coping strategy (Greenglass, 2002), student-teachers may get mentally prepared in advance and set appropriate expectations for the courses.

A limitation of the study is the length. Although one semester may not be a short period, a longer period of study may have resulted in more significant changes in the participants' emotions and professional identity. In future studies, documenting the continuum of student-teachers' learning experiences at university, during teaching practicum, and in their first few years of work may yield different results and reveal changes over time in a more complex and dynamic system. Another way in which future research can enrich our understanding of preservice teachers' emotions and professional identity development is to develop validated scales and conduct inferential analysis to statistically verify the relationships between the two.

## Data Availability Statement

The original contributions presented in the study are included in the article/Supplementary Material, further inquiries can be directed to the corresponding author/s.

## Ethics Statement

The studies involving human participants were reviewed and approved by the Professor Committee of the School of Foreign Languages and Literature Beijing Normal University. The patients/participants provided their written informed consent to participate in this study.

## Author Contributions

ZC contributed in conceptualization, methodology design, data analysis, drafts, and revision. YS contributed in instrument design, data analysis, drafts, and revision. ZJ helped with instrument design, data collection, and data analysis. All authors contributed to the article and approved the submitted version.

## Conflict of Interest

The authors declare that the research was conducted in the absence of any commercial or financial relationships that could be construed as a potential conflict of interest.

## Publisher's Note

All claims expressed in this article are solely those of the authors and do not necessarily represent those of their affiliated organizations, or those of the publisher, the editors and the reviewers. Any product that may be evaluated in this article, or claim that may be made by its manufacturer, is not guaranteed or endorsed by the publisher.

## Footnotes

^1^These students should work as teachers for certain number of years after graduation.
