# Supplementary material for: A Study of Student-Teachers' Emotional Experiences and Their Development of Professional Identities
Source: Front Psychol. 2022 Jan 25;12:810146. doi: 10.3389/fpsyg.2021.810146 (PMC8824257; doi:10.3389/fpsyg.2021.810146)
Supplement: Supplementary file 1 [file Data_Sheet_1.pdf]

Appendix I

Questionnaire of Student-teachers' Emotions

SECTION I: Background information

1. Grade: __________

2. ELT methodology related courses you took this semester:
○Language Learning Theories
○Curriculum and Teacher Materials
○ English Teaching Design
○English Language Teaching Methodology
○Action Research for English Teachers
Others: ____________________________
3. Gender: ○male ○female

SECTION II: Emotions experienced while learning those courses

1. I feel affection towards teaching after learning the courses.
2. I feel tired when I learn theories related to language learning and teaching.
3. Thinking of the possibility of my positive influence on my future students makes me happy.
4. Thinking of my future students evokes feelings of love inside me.
5. I think working with students in the future will make me frustrated.
6. Most of the time when I finished these courses I felt tired.
7. I feel nervous because I do not think I have the ability to help my future students to learn well.
8. Trying to apply theories in my teaching practice makes me feel excited.
9. I am happy when I design a lesson that I believe will help the potential students to learn.
10. I am proud that I can design a variety of effective teaching activities on my own.
11. When I receive positive feedback on my teaching design from my teacher, I feel that my confidence is growing.
12. Pride due to my classmates' positive feedback on my teaching design confirms to me that I am doing a good job.
13. I like motivating students to learn English.
14. I think lesson planning is tiring.
15. My practice confirms to me that being an English teacher is a good job.
16. After practicing, I feel I love my future job as an English teacher.
17. In my micro-teaching, I felt hopeless when some of the activities did not work.
18. When the teaching goals are not achieved in my micro-teaching, I feel frustrated.
19. When the class atmosphere in my micro-teaching is negative, I feel frustrated.
20. I feel hopeless when (choose no more than 3 items) ____________________
A. I cannot understand the theories
B. I cannot apply the theories
C. I find my teaching design does not work
D. I get negative feedback from the teacher
E. I get negative feedback from my classmates
F. I do not know how to improve my teaching design
G. Others ______________________
21. I feel more confident when (choose no more than 3 items)_________________
A. I can understand the theories
B. I can apply the theories
C. I find my teaching design work effectively
D. I get positive feedback from the teacher
E. I get positive feedback from my classmates
F. I figure out how to improve my teaching design
G. Others

Appendix II

Sample interview questions

(1) Have you experienced emotional changes during the ELT methodology-related courses? How did they influence your learning?

(2) What have you learned in these courses? How did you feel about your learning?
(3) Have you experienced any difficulties in the coursework? What were your emotions like? How did you deal with them?
(4) Is there any changes in your understanding of English teaching? Does your willingness to be a teacher increase or decrease? What leads to these changes?
(5) Can you share some critical events that triggered intense emotions during the coursework? What impacts did they have on you?

Appendix III

Reflection sheet (English version)

Have you experienced some emotions while taking ELT methodology-related courses last semester? How did you regulate them? Do you have a different understanding of English teaching after taking these courses? Please complete this reflection sheet based on your experiences. There is no correct or wrong answer and your responses will not influence your course assessment.

At the beginning of last semester, when I started to learn these courses, the emotions I felt were_______________. Among them, _______________(one kind of emotion) was the strongest because____. The primary effects of these emotions on me were____. I________ (did or didn't) try to regulate them by ____________________________________ (note: fill this blank with regulatory strategies you used when the emotions were negative).

During the semester, while doing assignments, teaching designs, and micro-teaching, the emotions I experienced were_____________________. Among them, _____________(one kind of emotion) was the strongest because____. The primary effects of these emotions on me were______________________________________. I________ (did or didn't) try to regulate them by _____________________________________ (note: fill this blank with regulatory strategies you used when the emotions were negative).

At the end of the semester, these courses finished. The emotions I had were________________. The most intense one was _____________because____. The primary effects of these emotions on me were____. I________(did or didn't) try to regulate them by ____________________________________ (note: fill this blank with regulatory strategies you used when the emotions were negative).
